# Associations of parental age at pregnancy with adolescent cognitive development and emotional and behavioural problems: a birth cohort in rural Western China

**DOI:** 10.1186/s12889-024-18309-z

**Published:** 2024-03-12

**Authors:** Wanting Wan, Yingze Zhu, Jiaxin Tian, Yue Cheng, Lingxia Zeng, Zhonghai Zhu

**Affiliations:** 1 Key Laboratory of Shaanxi Province for Craniofacial Precision Medicine Research, College of Stomatology, Xi’an Jiaotong University, Xi’an, 710049 Shaanxi P. R. China; 2 Department of Epidemiology and Biostatistics, School of Public Health, Xi’an Jiaotong University Health Science Center, No. 76, Yanta West Road, Xi’an, 710061 Shaanxi P. R. China; 3 Department of Nutrition and Food Safety Research, School of Public Health, Xi’an Jiaotong University Health Science Center, Xi’an, 710061 Shaanxi P. R. China; 4 Center for Chronic Disease Control and Prevention, Global Health Institution, Xi’an Jiaotong University, Xi’an, 710049 Shaanxi P. R. China; 5 Key Laboratory for Disease Prevention and Control and Health Promotion of Shaanxi Province, Xi’an, 710061 Shaanxi P. R. China

**Keywords:** Parental age, Early adolescence, Cognitive development, Emotional and behavioural problems, Birth cohort

## Abstract

**Background:**

The relationship between parental age at pregnancy and offspring development in low- and middle-income countries remains unclear. We aimed to examine the associations of parental age at pregnancy with adolescent development in rural China.

**Methods:**

We conducted a prospective birth cohort study of offspring born to pregnant women who participated in an antenatal micronutrient supplementation trial in rural Western China. Adolescent cognitive development and emotional and behavioural problems were assessed by using the Wechsler Intelligence Scale for Children-IV and the Youth Self-Report-2001, respectively. After accounting for the possible nonlinear relationships, we examined the linear associations between parental age (in years) at pregnancy and scores of adolescent cognitive development and emotional and behavioural problems by performing generalized estimating equations.

**Results:**

Among 1897 adolescents followed from birth to early adolescence, 59.5% were male with a mean age of 11.8 (standard deviation (SD): 0.8) years. The mean ages of mothers and fathers at pregnancy were 24.6 (SD: 4.4) and 27.9 (SD: 4.1) years old, respectively. All the *P* values of the nonlinear terms between parental age and adolescent development in all domains were greater than 0.05. Each one-year increase in maternal age at pregnancy was associated with a 0.29-point (95% confidence interval (CI) 0.06, 0.52) increase in the full-scale intelligence quotient in early adolescence. After parental age was categorized into quartiles, the total behavioural problem scores of adolescents with fathers with an age in the fourth quartile (Q4) were 6.71 (95% CI 0.86, 12.57) points higher than those of adolescents with fathers with an age in the first quartile (Q1), with a linear trend *P* value of 0.01. Similarly, higher scores (worse behavioural problems) were observed for internalizing behavioural problems and other emotional and behavioural symptoms related to anxiety, withdrawal, social problems, thought problems and aggressive behaviour.

**Conclusions:**

At conception, older maternal age was independently linked to better adolescent cognitive development, whereas advanced paternal age was independently associated with a greater risk of adolescent emotional and behavioral problems. These findings suggest that public health policies targeting an optimal parental age at pregnancy should be developed in the context of offspring developmental consequences.

**Supplementary Information:**

The online version contains supplementary material available at 10.1186/s12889-024-18309-z.

## Background

The average age of parents at first childbirth has dramatically increased in recent decades, particularly in developed settings [1]. Khandwala and colleagues analysed 168 million live births in the USA from 1972 to 2015 and reported that the mean paternal age increased from 27.4 to 30.9 years over the past 44 years [2]. This age increase with delayed childbearing is much greater among mothers [3], with similar tendencies in Nordic countries since the 1980s [4]. In addition to delayed childbearing in low- and middle-income countries (LMICs) [5], adolescent mothers becoming pregnant at less than 20 years of age is still a public health issue. As estimated in 2019, there were 21 million pregnancies among adolescents aged 15–19 years in LMICs each year, resulting in 12 million births [6]. In addition, a pooled analysis of data from 254 nationally representative demographic and health surveys including 747 thousand adolescents (aged 15–19 years) among 74 LMICs between 1990 and 2018 showed a slow reduction or even an increase in the prevalence of adolescent births [7].

Both young (including adolescence) and advanced maternal age were associated with increased rates of neonatal mortality and adverse birth outcomes [8, 9], which may impair the long-term neurodevelopment of the offspring. However, a brain imaging study from U.S. documented that children born to mothers of older age at conception had higher volumes in cortical regions, suggesting potential benefits on cognitive development [10]. In addition, studies from rodent models reported that increasing paternal age was linked to higher risk of germline de novo mutations and a modified sperm epigenome, which were enriched for embryo development and neurodevelopment [11]. These mechanisms suggest potentially different effects of advanced maternal and paternal age at pregnancy on offspring developmental outcomes. However, in epidemiological studies mainly focusing on a generally healthy population, it remains unclear whether parental age at pregnancy has a direct relationship with the long-term cognitive development and emotional and behavioural health in offspring.

Studies from developed countries reported that offspring born to teenage mothers had lower intelligence test scores than did those born to adult mothers, while this relationship was not observed for offspring born to teenage fathers after accounting for maternal age [10, 12]. In addition, Duncan and colleagues analysed data from the U.S. National Longitudinal Study of Youth and reported that each year of delayed first childbirth in mothers was associated with a 0.02 to 0.04 standard deviation (SD) increase in school achievement [13]. These results support examining the association between parental ages across a broader range and child cognitive development, including possible nonlinear relationships. For instance, both young and advanced parental age at conception may have consequences on offspring neurodevelopment.

A meta-analysis including 133,585 participants in 18 studies, all of which were conducted in developed countries, reported a higher risk of externalizing behaviour problems among children born to teenage mothers [14]. Nevertheless, a negative to null association between increasing maternal age and emotional and behavioural outcomes was found in Dutch population-based cohorts and a Spanish cohort [15, 16]. Similar inconsistent associations between paternal age and child developmental outcomes have been documented in developed settings [10, 15]. Therefore, these results suggest that providing novel evidence for public health interventions and policies to examine the possible nonlinear associations between parental age at pregnancy and long-term developmental outcomes in offspring in LMICs would be meaningful.

In the present study, we analysed data from a birth cohort of adolescents born to women who participated in a community micronutrient supplementation trial in rural Western China where the national socio-economy has been rapidly developing for decades. Over the past two decades in our study setting, as urbanization has been processing, the prevalence of child emotional and behavioural problems is substantially increasing especially among left-behind children, which is coupled with suboptimal cognitive development due to perinatal deprivations [17–19]. These early-life developmental challenges have been reported to be associated with a smaller number of completed school years, long-term compromised mental health in adulthood and an even lower risk of life-course cardiovascular diseases and mortality [20–23]. Here, we aimed to examine the associations of parental age at pregnancy with adolescent cognitive development and emotional and behavioural problems after we accounted for the nonlinear relationships in the rural areas of Western China.

## Methods

### Data source

We used data from a prospective birth cohort of offspring born to pregnant women who participated in a randomized controlled trial of micronutrient supplementation during pregnancy between 2002 and 2006. Pregnant women at less than 28 weeks gestation from each village in two counties in rural Western China were randomized to receive folic acid, iron/folic acid, or multiple micronutrients daily until delivery. A total of 4488 singleton infants from the parent trial were eligible for visits during early adolescence after excluding infants with birth defects, stillborn infants, and/or infants who died during early life. A total of 2118 adolescents aged 10–14 years were followed in 2016, and among them, 1897 completed the cognitive development and emotional and behavioural problem assessments (see Supplementary Fig. 1, Additional File 1). The procedures of the parent trial and follow-up study were previously described in detail [19, 24]. The trial and follow-up study were approved by the Ethics Committee of Xi’an Jiaotong University Health Science Center. Written informed consent was obtained from all mothers or primary caregivers, and oral assent was obtained from all adolescents.

### Measurements

#### Assessment of adolescent cognitive development

Adolescent cognitive development was assessed by standardly trained public health graduates using the Wechsler Intelligence Scale for Children, fourth edition (WISC-IV). This scale, comprising ten subtests (Block Design, Similarities, Digit Span, Picture Concepts, Coding, Vocabulary, Letter-Number Sequencing, Matrix Reasoning, Comprehension, and Symbol Search), along with four supplementary subtests (Picture Completion, Cancellation, Information, and Arithmetic), was administered face-to-face in a local school classroom with a quiet and comfortable environment. The assessments took place during the adolescence-stage visit between June and December 2016. Each adolescent was given only one opportunity to complete these assignments, even if they chose to discontinue or refused participation during the administration process, which lasted for approximately 1–2 h. This approach avoided test familiarity from repeated visits. The time the adolescent used to finish specific tests was counted to derive the level of cognitive development. Chen and colleagues locally adapted the WISC-IV using a representative sample which accounted for the population structure on sex, race, parent education level and geographic region of Chinese children [25]. Based on the Chinese norm of the WISC-IV, the adolescent age-standard full-scale intelligence quotient (FSIQ), which represents general cognitive development and other aspects of cognitive development, including verbal comprehension, perceptual reasoning, working memory and processing speed index, were derived. Higher test score indicates better adolescent cognitive development.

#### Assessment of adolescent emotional and behavioural problems

Adolescent emotional and behavioural problems were assessed by the Youth Self-Report (YSR-2001), which was completed by the adolescents themselves under standard instructions in a local school classroom without time restriction. The YSR-2001 has been locally adapted, validated and previously used in China [26]. The Chinese version was translated into Chinese and back-translated into English for cross-checking in the field. It underwent revisions by a panel of psychologist/psychiatrists, resulting in good-to-excellent intraclass correlation between 0.66 and 0.87 (median 0.83) and comparable area under the curve between 0.73 and 0.96 (median 0.90). These metrics indicated acceptable predictive validity for clinical outcomes compared to the original English version and other scales, e.g., the Strengths and Difficulties Questionnaire. Based on the 118 items of the YSR-2001, eight indexes related to symptoms were derived: (i) anxiety/depression, (ii) withdrawal, (iii) somatic complaints, (iv) social problems, (v) thought problems, (vi) attention problems, (vii) delinquent/rule-breaking behaviour and (viii) aggressive behaviour. Furthermore, the total behavioural problem score, accounting for all eight indexes; the internalizing problem score, accounting for Indexes i, ii and iii; and the externalizing problem score, accounting for Indexes vii and viii, were constructed by the YSR-2001. Higher test scores were indicative of greater or worse emotional and behavioural problems (negative outcome).

#### Parental age at pregnancy and potential confounders

Data on covariables were collected by trained public health graduates using face-to-face interviews at enrolment in the parent trial and at the early adolescence visit. Parental age (in years) at pregnancy was calculated by using the date of birth and the interview date at enrolment in the parent trial. We considered the following confounders: parental educational attainment (did not complete primary school;<3 years of schooling; primary school; secondary school; high school diploma and above), parental occupation type (farmer, other), household wealth, maternal mid-upper arm circumference at enrolment (< 21.5 cm, ≥ 21.5 cm), parity (0, ≥ 1), birthweight-for-gestational-age z score, adolescent sex (male, female), school type (village, town, county) and adolescent age. Parity is defined as the number of times a woman giving birth at 20 gestational weeks or more, regardless of whether the new born is livebirth or stillbirth. The household wealth index was constructed by using principal component analysis based on an inventory of 17 household assets, including ownership of goats, cattle, horses and poultry, cars, and dwelling characteristics [27]. The resulting continuous factor score was then utilized in the statistical analyses below. Birthweight-for-gestational-age z scores were established according to the INTERGROWTH-21st reference [28].

### Statistical analyses

We use numbers with percentages and means with SDs to describe the categorical and continuous variables, respectively. A two-sided *P* value less than 0.05 was considered to indicate statistical significance. All analyses were conducted with STATA 16.0 software.

In the present study, we included the FSIQ score and the total behavioural problem and internalizing and externalizing behavioural problem scores as the primary developmental outcomes and other aspects of cognitive development and emotional and behavioural problems among adolescents as the secondary outcomes. We first examined the possible nonlinear relationships between maternal and paternal age at pregnancy and adolescent primary developmental outcomes by using restricted cubic splines with four knots. As shown in Supplementary Fig. 2 (see Additional File 1), the shapes of the associations between maternal age and adolescent developmental outcomes were linear, with all *P* values of the nonlinear terms being greater than 0.1. Although the shapes of the associations between paternal age and adolescent emotional and behavioural outcomes showed nonlinearity, all the *P* values of the nonlinear terms test were greater than 0.05.

Therefore, we examined the linear associations between parental age (in years) and adolescent developmental outcomes. For general interest, we also presented the results categorizing parental age into quartiles, which could partly account for the nonlinear relationship that did not reach statistical significance potentially for a limited sample size in the present study. In all the models, we adjusted for parental educational attainment and occupation type, maternal parity and mid-upper arm circumference, randomized regimens, birthweight-for-gestational-age z score, household wealth in early adolescence, adolescent sex, height-for-age z score, school type and adolescent age, which have been reported to be associated with adolescent developmental outcomes [18, 19, 29]. In addition, parental age, adolescent FSIQ score and total behavioural problem score were mutually adjusted in all models, in all which the variance inflation factors (< 5) indicated no significant collinearity issues. Furthermore, the interaction term between maternal and paternal age did not reach statistical significance in all models. The adjusted mean differences and their 95% confidence intervals (95% CIs) were estimated by performing generalized estimating equations with identity link and exchangeable correlation structures.

For sensitivity analyses, to examine the potential for selection bias owing to loss to follow-up, we compared the baseline characteristics between adolescents who were followed up and completed the assessments and those who were lost to follow-up. In addition, we conducted inverse probability censor weighting to estimate the influence of this selection bias on adolescent primary developmental outcomes.

## Results

### Baseline characteristics

A total of 1897 participants were included in the final analysis. Table 1 presents the participants’ baseline characteristics. The mean ages of mothers and fathers at pregnancy were 24.6 (SD, 4.4; median, 24; interquartile range (IQR), 21–28) and 27.9 (SD, 4.1; median, 27; IQR, 25–31) years, respectively. The majority of parents had a secondary education level and were farmers. Among the 1897 adolescents, 59.5% were male, with a mean age of 11.8 (SD, 0.8; median, 12; IQR, 11–12) years old. A total of 4.0%, 3.3% and 13.8% of the adolescents were born prematurely, had a low birth weight and were small-for-gestational age at birth, respectively. In early adolescence, 2.3% and 14.6% of the adolescents had stunting and were overweight/obese, respectively.

**Table 1:** Background characteristics of the participants (*n* = 1897)

Parental and household characteristics	n (%)^a^	Adolescent characteristics	n (%)^a^
Maternal age (years) at pregnancy		Sex	
Mean (SD)	24.6(4.4)	Male	1129(59.5)
Median (IQR)	24(21–28)	Female	768(40.5)
Q1 (15 ~ 21)	561(29.6)	Birthweight (gram)/Mean (SD)	3211(415)
Q2 (22 ~ 24)	504(26.6)	Gestational weeks at delivery/Mean (SD)	39.8(1.6)
Q3 (25 ~ 28)	428(22.6)	Birthweight-for-gestational-age and sex z score/Mean (SD)	-0.19(1.04)
Q4 (29 ~ 41)	404(21.3)	Preterm (< 37 gestational weeks)	75(4.0)
Maternal education		Low birth weight (< 2500 g)	60(3.3)
<3 years	117(6.2)	Small-for-gestational age (< population 10th percentile)	245(13.8)
Primary	543(28.7)	Age (years)	
Secondary	975(51.5)	Mean (SD)	11.8(0.8)
High school and above	257(13.6)	Median (IQR)	12(11–12)
Maternal occupation		HAZ/Mean (SD)	0.1(1.1)
Farmer	1609(85.3)	BAZ/Mean (SD)	-0.22(1.1)
Other	278(14.7)	Overweight/obesity (BAZ ≥ 1 SD)	273(14.6)
Paternal age (years) at pregnancy		Stunting (HAZ<-2 SD)	44(2.3)
Mean (SD)	27.9(4.1)	School type	
Median (IQR)	27(25–31)	Village	272(14.3)
Q1 (20 ~ 25)	673(35.6)	Township	818(43.1)
Q2 (26 ~ 27)	345(18.2)	County	807(42.5)
Q3 (28 ~ 31)	478(25.3)	Household wealth at enrolment	
Q4 (32 ~ 44)	396(20.9)	Low	673(35.5)
Paternal education		Medium	602(31.7)
<3 years	27(1.4)	High	622(32.8)
Primary	287(15.2)	Adolescent cognitive test scores/Mean (SD)	
Secondary	1123(59.3)	Full-scale intelligence quotient	97.4(12.1)
High school and above	456(24.1)	Verbal comprehension index	102.1(15.4)
Paternal occupation		Working memory index	94.0(10.9)
Farmer	1441(76.1)	Perceptual reasoning index	95.9(12.0)
Other	452(23.9)	Processing speed index	99.2(13.6)
Parity at enrolment		Scores of adolescent emotional and behavioural problems/Mean (SD)	
0	1237(65.2)	Total problem score	49.5(25.5)
≥ 1	660(34.8)	Internalizing problem score	11.3(8.0)
Maternal mid-upper-arm circumference (cm) at enrolment/Mean (SD)		Externalizing problem score	8.6(7.5)
<21.5	331(17.6)	Anxiety/depression	4.8(3.9)
≥ 21.5	1547(82.4)	Withdrawn	3.4(2.7)
Household wealth at enrolment		Somatic complaints	3.1(3.0)
Low	629(33.2)	Social problems	4.0(3.3)
Medium	692(36.5)	Thought problems	2.8(2.9)
High	576(30.4)	Attention problems	4.4(2.9)
Randomized regimen		Rule breaking	2.8(3.1)
Folic acid	661(34.8)	Aggressive behaviour	5.9(4.9)
Folic acid plus iron	610(32.2)		
Multiple micronutrients	626(33.0)		

Abbreviations: BAZ, BMI-for-age and sex z score; BMI, body mass index; HAZ, height-for-age and sex z score; Interquartile range, IQR; SD, standard deviation

^a^Data are missing for maternal educational attainment (*n* = 5), maternal occupation type (*n* = 10), paternal age (*n* = 5), paternal educational attainment (*n* = 4), paternal occupation type (*n* = 4), maternal mid-upper-arm circumference (*n* = 19), birth weight (*n* = 87), birth weight-for-gestational-age and sex z score (*n* = 123), small-for-gestational age (*n* = 123), and adolescent height-for-age z score (*n* = 5) and BMI-for-age z score (*n* = 21)

### Maternal age at pregnancy and adolescent development

We found that maternal age at pregnancy was linearly associated with adolescent cognitive development (Table 2). Each one-year increase in maternal age at pregnancy was associated with a 0.29 (95% CI 0.06, 0.52) point increase in the FSIQ in offspring at early adolescence. Furthermore, for adolescents born to mothers with an age in the fourth quartile (Q4) during pregnancy, the FSIQ was 3.53 (95% CI 1.01, 6.06) points higher than that for adolescents born to mothers with an age in the first quartile (Q1), with a linear trend *P* value of 0.009. Similar positive associations were observed for verbal comprehension and the processing speed index (see Supplementary Table 1, Additional File 1). All the 95% CIs estimating the relationships between maternal age at pregnancy and adolescent emotional and behavioural problems crossed null points (Table 2 and see Supplementary Table 2, Additional File 1).

**Table 2:** Associations of maternal age at pregnancy with adolescent cognitive development and emotional and behavioural problems in a birth cohort in rural Western China. Higher scores indicated better cognitive development and worse emotional and behavioural problems

Test scores	Maternal age per year	Q1	Q2	Q3	Q4
FSIQ					
*n*	1897	561	504	428	404
Mean (SD)	97.4(12.1)	96.3(12.1)	99.0(12.1)	97.1(12.4)	97.3(11.6)
Adjusted mean differences (95% CI) ^a^	0.29(0.06, 0.52)	Reference	1.07(-0.35, 2.48)	1.66(-0.23, 3.56)	3.53(1.01, 6.06)
*P* value for linear trend	0.015	0.009			
Total behavioural problem score					
*n*	1897	561	504	428	404
Mean (SD)	49.5(25.5)	49.6(26.0)	48.3(24.9)	49.3(24.7)	51.1(26.5)
Adjusted mean differences (95% CI) ^a^	-0.08(-0.63, 0.46)	Reference	-0.88(-4.18, 2.41)	-2.68(-7.10, 1.74)	-2.03(-7.93, 3.87)
*P* value for linear trend	0.765	0.369			
Internalizing behavioural problem score					
*n*	1897	561	504	428	404
Mean (SD)	11.3(8.0)	11.2(8.3)	11.1(8.0)	11.0(7.5)	12.0(8.2)
Adjusted mean differences (95% CI) ^a^	0.02(-0.16, 0.19)	Reference	-0.88(-4.18, 2.41)	-2.68(-7.10, 1.74)	-2.03(-7.93, 3.87)
*P* value for linear trend	0.863	0.561			
Externalizing behavioural problem score					
*n*	1897	561	504	428	404
Mean (SD)	8.6(7.5)	8.5(7.5)	8.3(7.2)	8.7(7.3)	9.2(7.8)
Adjusted mean differences (95% CI) ^a^	-0.01(-0.17, 0.15)	Reference	-0.16(-1.12, 0.80)	-0.72(-2.00, 0.56)	-0.67(-2.38, 1.05)
*P* value for linear trend	0.898	0.364			

Abbreviations: CI, confidence interval; FSIQ, full-scale intelligence quotient; SD, standard deviation

^a^The adjustments included parental educational attainment and occupation type, paternal age, maternal parity and mid-upper arm circumference, randomized regimen, birthweight for gestational age z score, household wealth at early adolescence, adolescent sex, height-for-age z score, school type and total behavioural problem (for cognitive outcomes) or FSIQ (for emotional and behavioural outcomes)

### Paternal age at pregnancy and adolescent development

Interestingly, we did not observe consistent linear associations between paternal age at pregnancy and adolescent cognitive development (Table 3), except for the secondary outcome, i.e., the verbal comprehension index. A higher paternal age at pregnancy was associated with a lower verbal comprehension index score, with an adjusted mean difference of -0.39 (95% CI -0.72, -0.06) points for each one-year increase (see Supplementary Table 3, Additional File 1). The linear relationships between paternal age (in years) at pregnancy and adolescent emotional and behavioural problems were not statistically significant. However, adolescents with fathers with an age in the fourth quartile (Q4) at pregnancy had total behaviour problem scores that were 6.71 (95% CI 0.86, 12.57) points higher than those of adolescents with fathers with an age in the first quartile (Q1), with a linear trend *P* value of 0.01. Similarly, the adjusted mean difference for internalizing behavioural problems was 2.05 (95% CI 0.19, 3.91) points, and the linear trend *P* value was 0.035. Similar positive associations, i.e., older paternal age leading to a greater likelihood of behavioural problems, were observed for other aspects of adolescent emotional and behavioural problems, including anxiety, withdrawal, social problems, thought problems and aggressive behaviour (see Supplementary Table 4, Additional File 1).

**Table 3:** Associations of paternal age at pregnancy with adolescent cognitive development and emotional and behavioural problems in a birth cohort in rural Western China. Higher scores indicated better cognitive development and worse emotional and behavioural problems

Test scores	Paternal age per year	Q1	Q2	Q3	Q4
FSIQ					
*n*	1892	673	345	478	396
Mean (SD)	97.4(12.1)	98.2(12.1)	96.9(12.7)	97.3(11.9)	96.6(11.7)
Adjusted mean differences (95% CI) ^a^	-0.24(-0.48, 0.01)	Reference	-1.50(-3.00, 0.01)	-1.31(-3.06, 0.44)	-1.72(-4.24, 0.80)
*P* value for linear trend	0.064	0.097			
Total behavioural problem score					
*n*	1892	673	345	478	396
Mean (SD)	49.5(25.5)	47.8(25.3)	49.3(25.0)	50.8(25.5)	50.9(26.0)
Adjusted mean differences (95% CI) ^a^	0.45(-0.13, 1.03)	Reference	2.64(-0.86, 6.14)	5.07(1.00, 9.14)	6.71(0.86, 12.57)
*P* value for linear trend	0.126	0.010			
Internalizing behavioural problem score					
*n*	1892	673	345	478	396
Mean (SD)	11.3(8.0)	10.8(8.2)	11.1(8.0)	11.5(7.8)	12.1(8.0)
Adjusted mean differences (95% CI) ^a^	0.07(-0.12, 0.25)	Reference	0.50(-0.61, 1.61)	1.11(-0.18, 2.40)	2.05(0.19, 3.91)
*P* value for linear trend	0.467	0.035			
Externalizing behavioural problem score					
*n*	1892	673	345	478	396
Mean (SD)	8.6(7.4)	8.1(7.2)	8.5(7.2)	9.3(7.7)	8.9(7.5)
Adjusted mean differences (95% CI) ^a^	0.09(-0.08, 0.25)	Reference	0.60(-0.42, 1.61)	1.38(0.19, 2.56)	1.09(-0.62, 2.79)
*P* value for linear trend	0.317	0.062			

Abbreviations: CI, confidence interval; FSIQ, full-scale intelligence quotient; SD, standard deviation

^a^The adjustments included parental educational attainment and occupation type, maternal age, maternal parity and mid-upper arm circumference, randomized regimen, birthweight for gestational age z score, household wealth at early adolescence, adolescent sex, height-for-age z score, school type and total behavioural problem (for cognitive outcomes) or FSIQ (for emotional and behavioural outcomes)

### Sensitivity analyses

Supplementary Table 5 (see Additional File 1) shows that adolescents from families with better socioeconomic status, such as families with higher parental educational attainment and household wealth, were more likely to be lost to follow-up, with *P* values less than 0.05. As shown in Supplementary Tables 6 and 7 (see Additional File 1), the results obtained using inverse probability censor weighting to account for selection bias due to loss to follow-up remained similar to those in Tables 2 and 3, and the 95% CIs were qualitatively unchanged.

## Discussion

In this prospective birth cohort study with a 14-year follow-up, we found that older maternal age at pregnancy was independently associated with better adolescent cognitive development but not with behavioural outcomes. In contrast, advanced paternal age at pregnancy was independently associated with a higher risk of adolescent emotional and behavioural problems.

By accounting for the possible nonlinearity of maternal age (in years) at pregnancy with adolescent developmental outcomes using an appropriate statistical method, our study expands the literature by showing that increasing maternal age has a linearly positive relationship with adolescent cognitive development [12]. In addition, this relationship remained unchanged after we adjusted for a much wider range of confounders, including family socioeconomic status, maternal reproductive history and nutritional status during periconception and adolescent birth outcomes, physical status and education status. To our knowledge, there are no previous studies that mutually adjusted for offspring cognitive development and behavioural problems, which could act as a surrogate for the commonly unmeasured caregiving environment in the literature.

The biological disadvantage of reproductive-aged women delaying their first pregnancies, raises concerns about offspring development in developed settings, such as a higher risk of pregnancy complications, older age-related epigenetic aberrations and foetal chromosomal abnormalities [30]. However, in rural areas of developing settings, our findings suggest that the benefits of a mother delaying her first pregnancy on adolescent long-term cognitive development may compensate for the biological disadvantages of increasing age within an appropriate range. It seems implausible to explain these benefits by the underlying biological mechanism, and they may be better explained by greater social support, better family functioning, nurturing environments, and a lower risk of family conflicts resulting from an older maternal and paternal age [31, 32]. Nevertheless, based on a brain imaging study of 8709 mothers aged 15–45 years from the Adolescent Brain Cognitive Development (ABCD) cohort in the U.S., Du and colleagues revealed that higher cortical region volumes mediated the linear associations between older maternal age and better cognitive performance and a lower risk of behavioural problems in children [10]. These findings suggest a possible causal link between increasing parental age and offspring developmental outcomes, warranting further investigations of the underlying biological mechanism involved. Although some studies have reported statistical associations of maternal age at pregnancy with offspring behavioural problems [33], we did not observe any statistically significant relationship after adjusting for paternal age. The discrepancies may be partly attributed to using maternal age in adolescence and young twenties as the control and not adjusting for the paternal age [33]. However, our findings agreed with those of another study conducted among 2894,688 participants in Denmark that focused on psychiatric disorders [34].

In contrast, we found consistently positive associations between older paternal age and higher adolescent emotional and behavioural problem scores. Worse or impaired emotional and behavioural development in early life and even small changes in emotional and behavioural problem scores have been shown to be associated with later mental health disorders [23]. Following this perspective, our findings are consistent with those of prior studies reporting a higher risk of different mental health disorders among offspring born to older fathers (> 35 years old) than among those born to fathers aged between 25 and 29 years [34]. Several biological mechanisms underlie this relationship. In animal studies, Milekic and colleagues reported a significant loss of methylation related to the transcriptional regulation of genes implicated in autism and schizophrenia in sperm samples from older mice, which resulted in similar DNA methylation abnormalities and disordered positive behaviours among mouse offspring [35]. Similar findings involving the expression of microRNAs and/or epigenetic changes, which are associated with regulating neuronal plasticity, brain morphological changes and frontohippocampal connectivity [36, 37], have been reported in other animal studies. These results suggest a possible causal link between older paternal age at pregnancy and long-term alterations in emotional and behavioural problems among offspring. In addition, several social factors may also play a role. We hypothesize that older fathers may be more likely to feel tired and stressed due to work and life than younger fathers are, possibly resulting in reduced interactions with children [38]. Taanila and colleagues reported that children who had good interactions with their parents had fewer behavioural problems [39]. The different associations between maternal and paternal age at pregnancy and adolescent cognitive development and emotional and behavioural problems have not been previously reported. We hypothesized that, in our study setting, mothers would usually take care of the child regardless of their age and communicate with the child, leading to equal distributions of adolescent behavioural problems across the range of maternal ages. In other words, adolescent cognitive development and emotional and behavioural problems may share somewhat different parent-offspring determinants. Taken together, these results suggest that the optimal parent age at pregnancy should be considered in the context of offspring developmental outcomes and that these findings may differ by setting.

### Implications in public health practices

Globally, the increase in the delay of childbearing age have become a significant public health issue, particularly over recent decades due to substantial socioeconomic development [1–4, 40, 41]. Given such a situation, it is crucial to consider parental age at pregnancy as a relevant factor when assessing children with behavioral or cognitive problems. In addition, these findings hold potential for meaningful implementations of public health policies aimed at optimizing parental age at pregnancy globally. This is particularly important for improving adolescent cognitive development and emotional and behavioral health, given their associations with better life-course human capital outcomes, a lower risk of cardiovascular diseases, and reduced adult mortality [20–23]. To address these issues effectively, public health programs should be developed and initiated to raise awareness among the population about suboptimal developmental risks among children born to parents having births outside the optimal childbearing age. Furthermore, early detection and monitoring of subtle developmental impairments in these infants would allow early intervention programs to avoid and prevent future cognitive defect and even neuropsychiatric disorders. Implementing these initiatives will, in turn, contribute significantly to achieving key Sustainable Development Goals (SDGs), notably SDG 1 (No poverty), SDG 2 (Zero hunger), SDG 3 (Good health and well-being), and SDG 4 (Quality education).

Our study has several limitations. First, we used data from a community trial in rural Western China, which may limit the generalization of our findings to other developed settings. Furthermore, our sample had few mothers with an age in the upper extreme range with a low percentage (0.8%) of mothers older than 35 years, which may explain the unobserved nonlinear relationships between very advanced maternal age and offspring cognitive development. Falster and colleagues reported a tendency towards an increased risk of cognitive developmental vulnerability (test score < population 10th percentile) among mothers older than 40 years at pregnancy [42]. Therefore, due to the tendency toward increasing maternal age at pregnancy in LMICs, a larger sample covering the full range of reproductive maternal ages in multiple regions is warranted to examine the relationship between maternal age at pregnancy and adolescent developmental outcomes. Third, as with other birth cohorts with long-term follow-ups, the participants lost to follow-up from pregnancy to early adolescence may raise the issue of selection bias. It seems plausible that adolescents from families with better socioeconomic status were more likely to be lost to follow-up, but the differences were not quantitatively noticeable. We adjusted for these unbalanced characteristics in the models. In addition, the results of sensitivity analyses using inverse probability censor weighting suggested the minimal influence of selection bias on our findings. Fourth, although we did not clearly clarify parental age at first pregnancy in our sample, this would be believable due to the single-child policy that was strictly implemented in recent decades in China. Fifth, in the present study, we were not able to ascertain whether advanced maternal and paternal ages at pregnancy exhibit shared mechanisms or pathways that are linked to long-term developmental outcomes in offspring. Understanding such shared mechanisms could inform promising public health interventions and programs to simultaneously reduce the developmental risks in offspring born to parents with advanced age. Finally, causal inference is naturally limited in studies with observational designs.

## Conclusions

We found that maternal age at pregnancy was linearly associated with better adolescent cognitive development, while paternal age was roughly linearly associated with a greater risk of emotional and behavioural problems in adolescents through the range of parental ages represented in the sample. Parental age at conception should be considered as part of the background when assessing children with behavioral or cognitive problems. Additionally, these findings suggest that public health policies and initiatives aiming for an optimal parental childbearing age should take into account the long-term developmental consequences among the next generations. Implementing of such strategies will eventually contribute significantly to achieving key SDGs.

### Electronic supplementary material

Below is the link to the electronic supplementary material.

Supplementary Material 1

## Data Availability

The datasets used and/or analysed during the current study are available from the corresponding author upon reasonable request.

## Abbreviations

LMICs: Low- and middle-income countries
WISC-IV: Wechsler Intelligence Scale for Children, fourth edition
FSIQ: Full-scale intelligence quotient
YSR: Youth Self-Report
SD: Standard deviation
CI: Confidence interval
ABCD: Adolescent Brain Cognitive Development
