# Supplementary material for: Associations of parental age at pregnancy with adolescent cognitive development and emotional and behavioural problems: a birth cohort in rural Western China
Source: BMC Public Health. 2024 Mar 12;24:775. doi: 10.1186/s12889-024-18309-z (PMC10935899; doi:10.1186/s12889-024-18309-z)

Supplementary Table 1 Associations of maternal age at pregnancy with other aspects of adolescent cognitive development in a birth cohort in rural western China. Higher scores indicate better cognitive development.

| Test scores | Maternal age per year | Q1 | *Q2* | *Q3* | *Q4* |
| --- | --- | --- | --- | --- | --- |
| VCI |  |  |  |  |  |
| *n* | 1897 | 561 | 504 | 428 | 404 |
| Mean (SD) | 102.1(15.4) | 100.6(15.2) | 104.0(15.5) | 101.0(16.0) | 102.8(14.7) |
| Adjusted mean differences (95% CI) ^a^ | 0.39(0.08, 0.70) | Reference | 1.84(-0.04, 3.73) | 1.29(-1.24, 3.81) | 5.25(1.88, 8.61) |
| *P* value for linear trend | 0.015 | 0.008 | | | |
| WMI |  |  |  |  |  |
| *n* | 1897 | 561 | 504 | 428 | 404 |
| Mean (SD) | 94.0(10.9) | 93.2(10.7) | 95.3(10.8) | 94.1(11.3) | 93.5(10.6) |
| Adjusted mean differences (95% CI) ^a^ | 0.14(-0.08, 0.36) | Reference | 1.08(-0.26, 2.42) | 1.56(-0.24, 3.35) | 1.83(-0.56, 4.23) |
| *P* value for linear trend | 0.224 | 0.081 | | | |
| PRI |  |  |  |  |  |
| *n* | 1897 | 561 | 504 | 428 | 404 |
| Mean (SD) | 95.9(12.0) | 95.2(12.7) | 97.3(11.4) | 96.0(12.0) | 94.8(11.5) |
| Adjusted mean differences (95% CI) ^a^ | 0.07(-0.17, 0.31) | Reference | 0.85(-0.62, 2.31) | 1.04(-0.93, 3.00) | 0.30(-2.32, 2.92) |
| *P* value for linear trend | 0.576 | 0.547 | | | |
| PSI |  |  |  |  |  |
| *n* | 1897 | 561 | 504 | 428 | 404 |
| Mean (SD) | 99.2(13.6) | 98.9(13.0) | 99.2(14.3) | 99.2(13.6) | 99.6(13.4) |
| Adjusted mean differences (95% CI) ^a^ | 0.30(0.02, 0.57) | Reference | -0.75(-2.42, 0.92) | 1.71(-0.53, 3.94) | 3.88(0.90, 6.86) |
| *P* value for linear trend | 0.034 | 0.033 | | | |

Abbreviation: CI, confidence interval; Perceptual reasoning index, PRI; Processing speed index, PSI; SD, standard deviation; Verbal comprehension index, VCI; Working memory index, WMI.

^a^The adjustments included parental education and occupation, paternal age, maternal parity and mid-upper arm circumference, randomized regimens, birthweight for gestational age z score, household wealth at early adolescence, and adolescent sex, height-for-age z score, school type and total behavioural problem (for cognitive outcome) or FSIQ (for emotional and behavioural outcome).

Supplementary Table 2 Associations of maternal age at pregnancy with adolescent other aspects of emotional and behavioural problems in a birth cohort in rural western China. Higher scores indicate worse emotional and behavioural problems.

| Test scores | Maternal age per year | Q1 | *Q2* | *Q3* | *Q4* |
| --- | --- | --- | --- | --- | --- |
| Anxiety/depression |  |  |  |  |  |
| *n* | 1897 | 561 | 504 | 428 | 404 |
| Mean (SD) | 4.8(3.9) | 4.7(3.9) | 4.8(3.9) | 4.7(3.8) | 5.2(4.1) |
| Adjusted mean differences (95% CI) ^a^ | 0.03(-0.05, 0.11) | Reference | 0.11(-0.40, 0.62) | -0.27(-0.94, 0.41) | 0.11(-0.79, 1.02) |
| *P* value for linear trend | 0.494 | 0.980 | | | |
| Withdrawn |  |  |  |  |  |
| *n* | 1897 | 561 | 504 | 428 | 404 |
| Mean (SD) | 3.4(2.7) | 3.5(2.8) | 3.2(2.6) | 3.4(2.5) | 3.6(2.7) |
| Adjusted mean differences (95% CI) ^a^ | -0.03(-0.08, 0.03) | Reference | -0.17(-0.52, 0.18) | -0.42(-0.89, 0.05) | -0.29(-0.91, 0.34) |
| *P* value for linear trend | 0.349 | 0.201 | | | |
| Somatic complaints |  |  |  |  |  |
| *n* | 1897 | 561 | 504 | 428 | 404 |
| Mean (SD) | 3.0(3.0) | 3.0(3.1) | 3.0(2.9) | 3.0(2.8) | 3.2(3.0) |
| Adjusted mean differences (95% CI) ^a^ | 0.01(-0.05, 0.08) | Reference | 0.13(-0.26, 0.51) | -0.25(-0.76, 0.27) | -0.08(-0.77, 0.61) |
| *P* value for linear trend | 0.677 | 0.699 | | | |
| Social problems |  |  |  |  |  |
| *n* | 1897 | 561 | 504 | 428 | 404 |
| Mean (SD) | 4.0(3.3) | 4.0(3.5) | 3.9(3.1) | 3.9(3.2) | 4.2(3.4) |
| Adjusted mean differences (95% CI) ^a^ | -0.03(-0.10, 0.04) | Reference | -0.05(-0.48, 0.37) | -0.45(-1.02, 0.11) | -0.46(-1.22, 0.30) |
| *P* value for linear trend | 0.367 | 0.181 | | | |
| Thought problems |  |  |  |  |  |
| *n* | 1897 | 561 | 504 | 428 | 404 |
| Mean (SD) | 2.8(3.1) | 2.7(2.8) | 2.8(3.0) | 2.7(2.8) | 2.9(3.0) |
| Adjusted mean differences (95% CI) ^a^ | -0.02(-0.08, 0.05) | Reference | 0.09(-0.29, 0.47) | -0.23(-0.74, 0.28) | -0.19(-0.88, 0.49) |
| *P* value for linear trend | 0.626 | 0.547 | | | |
| Attention problems |  |  |  |  |  |
| *n* | 1897 | 561 | 504 | 428 | 404 |
| Mean (SD) | 4.4(2.9) | 4.6(3.0) | 4.3(2.9) | 4.5(2.8) | 4.4(2.9) |
| Adjusted mean differences (95% CI) ^a^ | -0.01(-0.07, 0.05) | Reference | -0.14(-0.51, 0.24) | -0.22(-0.72, 0.28) | -0.25(-0.92, 0.42) |
| *P* value for linear trend | 0.762 | 0.398 | | | |
| Rule breaking |  |  |  |  |  |
| *n* | 1897 | 561 | 504 | 428 | 404 |
| Mean (SD) | 2.8(3.1) | 2.7(3.1) | 2.7(3.1) | 2.7(3.0) | 3.0(3.2) |
| Adjusted mean differences (95% CI) ^a^ | 0.01(-0.06, 0.07) | Reference | 0.11(-0.29, 0.51) | -0.19(-0.72, 0.34) | -0.10(-0.82, 0.61) |
| *P* value for linear trend | 0.806 | 0.720 | | | |
| Aggressive behavior |  |  |  |  |  |
| *n* | 1897 | 561 | 504 | 428 | 404 |
| Mean (SD) | 5.9(4.9) | 5.9(5.0) | 5.6(4.8) | 5.9(4.8) | 6.2(5.0) |
| Adjusted mean differences (95% CI) ^a^ | -0.02(-0.12, 0.09) | Reference | -0.27(-0.91, 0.36) | -0.53(-1.38, 0.32) | -0.56(-1.70, 0.58) |
| *P* value for linear trend | 0.728 | 0.252 | | | |

Abbreviation: CI, confidence interval; SD, standard deviation.

^a^The adjustments included parental education and occupation, paternal age, maternal parity and mid-upper arm circumference, randomized regimens, birthweight for gestational age z score, household wealth at early adolescence, and adolescent sex, height-for-age z score, school type and total behavioural problem (for cognitive outcome) or FSIQ (for emotional and behavioural outcome).

Supplementary Table 3 Associations of paternal age at pregnancy with other aspects of adolescent cognitive development in a birth cohort in rural western China. Higher scores indicate better cognitive development.

| Test scores | Paternal age per year | Q1 | *Q2* | *Q3* | *Q4* |
| --- | --- | --- | --- | --- | --- |
| VCI |  |  |  |  |  |
| *n* | 1892 | 673 | 345 | 478 | 396 |
| Mean (SD) | 102.1(15.4) | 102.9(15.4) | 101.2(15.9) | 102.2(15.4) | 101.4(14.7) |
| Adjusted mean differences (95% CI) ^a^ | -0.39(-0.72, -0.06) | Reference | -2.61(-4.62, -0.61) | -2.26(-4.60, 0.07) | -3.28(-6.64, 0.07) |
| *P* value for linear trend | 0.022 | 0.024 | | | |
| WMI |  |  |  |  |  |
| *n* | 1892 | 673 | 345 | 478 | 396 |
| Mean (SD) | 94.0(10.9) | 94.4(10.8) | 93.9(11.5) | 94.0(10.5) | 93.4(10.8) |
| Adjusted mean differences (95% CI) ^a^ | 0.01(-0.23, 0.25) | Reference | -0.43(-1.86, 1.00) | 0.36(-1.30, 2.02) | 0.33(-2.06, 2.72) |
| *P* value for linear trend | 0.928 | 0.786 | | | |
| PRI |  |  |  |  |  |
| *n* | 1892 | 673 | 345 | 478 | 396 |
| Mean (SD) | 95.9(12.0) | 96.3(11.9) | 96.2(12.7) | 96.0(12.0) | 94.8(11.6) |
| Adjusted mean differences (95% CI) ^a^ | -0.16(-0.42, 0.10) | Reference | -0.003(-1.56, 1.56) | -0.18(-2.00, 1.64) | -0.73(-3.34, 1.89) |
| *P* value for linear trend | 0.228 | 0.687 | | | |
| PSI |  |  |  |  |  |
| *n* | 1892 | 673 | 345 | 478 | 396 |
| Mean (SD) | 99.2(13.6) | 100.2(13.4) | 98.5(13.6) | 98.5(14.2) | 99.1(13.0) |
| Adjusted mean differences (95% CI) ^a^ | -0.14(-0.43, 0.15) | Reference | -1.47(-3.24, 0.31) | -1.96(-4.02, 0.11) | -1.07(-4.04, 1.91) |
| *P* value for linear trend | 0.352 | 0.156 | | | |

Abbreviation: CI, confidence interval; Perceptual reasoning index, PRI; Processing speed index, PSI; SD, standard deviation; Verbal comprehension index, VCI; Working memory index, WMI.

^a^The adjustments included parental education and occupation, maternal age, maternal parity and mid-upper arm circumference, randomized regimens, birthweight for gestational age z score, household wealth at early adolescence, and adolescent sex, height-for-age z score, school type and total behavioural problem (for cognitive outcome) or FSIQ (for emotional and behavioural outcome).

Supplementary Table 4 Associations of paternal age at pregnancy with adolescent other aspects of emotional and behavioural problems in a birth cohort in rural western China. Higher scores indicate worse emotional and behavioural problems.

| Test scores | Paternal age per year | Q1 | *Q2* | *Q3* | *Q4* |
| --- | --- | --- | --- | --- | --- |
| Anxiety/depression |  |  |  |  |  |
| *n* | 1892 | 673 | 345 | 478 | 396 |
| Mean (SD) | 4.8(3.9) | 4.6(3.8) | 4.9(3.9) | 5.0(3.9) | 5.1(4.0) |
| Adjusted mean differences (95% CI) ^a^ | 0.02(-0.07, 0.11) | Reference | 0.40(-0.14, 0.94) | 0.62(-0.01, 1.24) | 0.84(-0.06, 1.74) |
| *P* value for linear trend | 0.682 | 0.037 | | | |
| Withdrawn |  |  |  |  |  |
| *n* | 1892 | 673 | 345 | 478 | 396 |
| Mean (SD) | 3.4(2.7) | 3.3(2.7) | 3.3(2.7) | 3.4(2.5) | 3.6(2.7) |
| Adjusted mean differences (95% CI) ^a^ | 0.04(-0.02, 0.10) | Reference | 0.17(-0.20, 0.54) | 0.31(-0.12, 0.74) | 0.72(0.10, 1.34) |
| *P* value for linear trend | 0.175 | 0.040 | | | |
| Somatic complaints |  |  |  |  |  |
| *n* | 1892 | 673 | 345 | 478 | 396 |
| Mean (SD) | 3.0(3.0) | 2.9(2.9) | 2.9(2.9) | 3.1(2.9) | 3.4(3.0) |
| Adjusted mean differences (95% CI) ^a^ | 0.01(-0.06, 0.07) | Reference | -0.08(-0.48, 0.33) | 0.18(-0.29, 0.66) | 0.49(-0.19, 1.18) |
| *P* value for linear trend | 0.836 | 0.265 | | | |
| Social problems |  |  |  |  |  |
| *n* | 1892 | 673 | 345 | 478 | 396 |
| Mean (SD) | 4.0(3.3) | 3.7(3.2) | 4.1(3.5) | 4.2(3.2) | 4.2(3.3) |
| Adjusted mean differences (95% CI) ^a^ | 0.10(0.02, 0.17) | Reference | 0.47(0.02, 0.93) | 0.72(0.20, 1.24) | 1.06(0.30, 1.81) |
| *P* value for linear trend | 0.009 | 0.002 | | | |
| Thought problems |  |  |  |  |  |
| *n* | 1892 | 673 | 345 | 478 | 396 |
| Mean (SD) | 2.8(2.9) | 2.6(2.8) | 2.7(2.7) | 2.9(3.1) | 2.9(3.0) |
| Adjusted mean differences (95% CI) ^a^ | 0.05(-0.02, 0.11) | Reference | 0.23(-0.17, 0.64) | 0.53(0.06, 1.00) | 0.72(0.04, 1.40) |
| *P* value for linear trend | 0.178 | 0.021 | | | |
| Attention problems |  |  |  |  |  |
| *n* | 1892 | 673 | 345 | 478 | 396 |
| Mean (SD) | 4.4(2.9) | 4.3(3.0) | 4.6(3.0) | 4.5(2.7) | 4.4(3.0) |
| Adjusted mean differences (95% CI) ^a^ | 0.02(-0.05, 0.09) | Reference | 0.32(-0.07, 0.72) | 0.26(-0.20, 0.72) | 0.40(-0.27, 1.07) |
| *P* value for linear trend | 0.570 | 0.173 | | | |
| Rule breaking |  |  |  |  |  |
| *n* | 1892 | 673 | 345 | 478 | 396 |
| Mean (SD) | 2.7(3.1) | 2.5(3.0) | 2.7(3.0) | 3.0(3.3) | 2.9(3.1) |
| Adjusted mean differences (95% CI) ^a^ | 0.01(-0.06, 0.08) | Reference | 0.17(-0.26, 0.59) | 0.47(-0.02, 0.96) | 0.20(-0.51, 0.90) |
| *P* value for linear trend | 0.795 | 0.218 | | | |
| Aggressive behavior |  |  |  |  |  |
| *n* | 1892 | 673 | 345 | 478 | 396 |
| Mean (SD) | 5.9(4.9) | 5.5(4.9) | 5.8(4.9) | 6.3(5.0) | 6.1(4.9) |
| Adjusted mean differences (95% CI) ^a^ | 0.08(-0.03, 0.19) | Reference | 0.43(-0.25, 1.10) | 0.91(0.12, 1.69) | 0.89(-0.242.02) |
| *P* value for linear trend | 0.178 | 0.041 | | | |

Abbreviation: CI, confidence interval; SD, standard deviation.

^a^The adjustments included parental education and occupation, maternal age, maternal parity and mid-upper arm circumference, randomized regimens, birthweight for gestational age z score, household wealth at early adolescence, and adolescent sex, height-for-age z score, school type and total behavioural problem (for cognitive outcome) or FSIQ (for emotional and behavioural outcome).

Supplementary Table 5 Comparisons of baseline characteristics between adolescents who completed the assessments and those who did not complete the assessments.

| Baseline characteristics | Adolescents who completed the assessments/ *n*(%) | Adolescents who did not complete the assessments/ *n*(%) | *P* value |
| --- | --- | --- | --- |
| *n* | 1897(42.3) | 2591(57.7) |  |
| Maternal age (years)/mean (SD) | 24.6(4.4) | 24.6(4.4) | 0.593 |
| Q1 (15~21) | 561(29.6) | 57(26.3) | 0.118 |
| Q2 (22~24) | 504(26.6) | 74(34.1) |  |
| Q3 (25~28) | 428(22.6) | 47(21.7) |  |
| Q4 (29~41) | 404(21.3) | 39(18) |  |
| Maternal education |  |  | 0.002 |
| ＜3 years | 117(6.2) | 133(5.2) |  |
| Primary | 543(28.7) | 626(24.3) |  |
| Secondary | 975(51.5) | 1436(55.7) |  |
| High school and above | 257(13.6) | 383(14.9) |  |
| Maternal occupation |  |  | 0.035 |
| Farmer | 1609(85.3) | 2131(82.9) |  |
| Others | 278(14.7) | 439(17.1) |  |
| Paternal age (years)/ Mean (SD) | 27.9(4.1) | 27.8(4.1) | 0.721 |
| Q1 (20~25) | 673(35.6) | 81(37.5) | 0.856 |
| Q2 (26~27) | 345(18.2) | 42(19.4) |  |
| Q3 (28~31) | 478(25.3) | 51(23.6) |  |
| Q4 (32~44) | 396(20.9) | 42(19.4) |  |
| Paternal education |  |  | 0.001 |
| ＜3 years | 27(1.4) | 33(1.3) |  |
| Primary | 287(15.2) | 288(11.2) |  |
| Secondary | 1123(59.3) | 1645(63.8) |  |
| High school and above | 456(24.1) | 611(23.7) |  |
| Paternal occupation |  |  | 0.102 |
| Farmer | 1441(76.1) | 1910(74) |  |
| Others | 452(23.9) | 672(26) |  |
| Parity at enrollment |  |  | 0.947 |
| 0 | 1237(65.2) | 1692(65.3) |  |
| ≥1 | 660(34.8) | 899(34.7) |  |
| Maternal mid-upper-arm circumference (cm) at enrollment/Mean (SD) |  |  | 0.408 |
| ＜21.5 | 331(17.6) | 476(18.6) |  |
| ≥21.5 | 1547(82.4) | 2084(81.4) |  |
| Household wealth at enrollment |  |  | 0.004 |
| Low | 629(33.2) | 804(31) |  |
| Medium | 692(36.5) | 877(33.9) |  |
| High | 576(30.4) | 910(35.1) |  |
| Randomized regimens |  |  | 0.402 |
| Folic acid | 661(34.8) | 924(35.7) |  |
| Folic acid plus iron | 610(32.2) | 861(33.2) |  |
| Multiple micronutrients | 626(33.0) | 806(31.1) |  |
| Sex |  |  | <0.001 |
| Male | 1129(59.5) | 1362(52.6) |  |
| Female | 768(40.5) | 1229(47.4) |  |
| Birthweight (gram)/Mean (SD) | 3211(415) | 3211(415) | 0.054 |
| Gestational weeks at delivery/Mean (SD) | 39.8(1.6) | 39.8(1.6) | 0.651 |
| Birthweight-for-gestational-age and sex z-score/Mean (SD) | -0.19(1.04) | -0.20(1.00) | 0.300 |
| Preterm (< 37 gestational weeks) | 75(4.0) | 133(5.1) | 0.063 |
| Low birth weight (< 2500 g) | 60(3.3) | 86(3.6) | 0.677 |
| Small-for-gestational age (< population 10^th^ percentile) | 245(13.8) | 361(15.3) | 0.181 |

Abbreviations: SD, standard deviation.

Supplementary Table 6 Sensitivity analysis using inverse probability of censoring weighting to assess the potential of loss to follow-up on affecting estimates of associations of maternal age at pregnancy with adolescent cognitive development and emotional and behavioural problems in a birth cohort in rural western China. Higher scores indicate better cognitive development and worse emotional and behavioural problems.

| Test scores | Maternal age per year | Q1 | *Q2* | *Q3* | *Q4* |
| --- | --- | --- | --- | --- | --- |
| FSIQ |  |  |  |  |  |
| Adjusted mean differences (95% CI) ^a^ | 0.29(0.05, 0.53) | Reference | 1.10(-0.13, 2.33) | 1.68(0.31, 3.05) | 3.55(0.97, 6.14) |
| *P* value for linear trend | 0.019 | 0.003 | | | |
| Total behavioural problem |  |  |  |  |  |
| Adjusted mean differences (95% CI) ^a^ | -0.07(-0.60, 0.45) | Reference | -0.93(-4.39, 2.53) | -2.35(-6.50, 1.79) | -1.77(-7.91, 4.37) |
| *P* value for linear trend | 0.785 | 0.431 | | | |
| Internalizing behavioural problem |  |  |  |  |  |
| Adjusted mean differences (95% CI) ^a^ | 0.02(-0.14, 0.19) | Reference | 0.02(-1.09, 1.12) | -0.86(-2.17, 0.46) | -0.17 (-2.02, 1.68) |
| *P* value for linear trend | 0.804 | 0.613 | | | |
| Externalizing behavioural problem |  |  |  |  |  |
| Adjusted mean differences (95% CI) ^a^ | -0.01(-0.16, 0.15) | Reference | -0.16(-1.15, 0.83) | -0.63(-1.80, 0.54) | -0.57(-2.33, 1.18) |
| *P* value for linear trend | 0.937 | 0.421 | | | |

Abbreviation: CI, confidence interval; FSIQ, full-scale intelligent quotient.

^a^The adjustments included parental education and occupation, paternal age, maternal parity and mid-upper arm circumference, randomized regimens, birthweight for gestational age z score, household wealth at early adolescence, and adolescent sex, height-for-age z score, school type and total behavioural problem (for cognitive outcome) or FSIQ (for emotional and behavioural outcome).

Supplementary Table 7 Sensitivity analysis using inverse probability of censoring weighting to assess the potential of loss to follow-up on affecting estimates of associations of paternal age at pregnancy with adolescent cognitive development and emotional and behavioural problems in a birth cohort in rural western China. Higher scores indicate better cognitive development and worse emotional and behavioural problems.

| Test scores | Paternal age per year | Q1 | *Q2* | *Q3* | *Q4* |
| --- | --- | --- | --- | --- | --- |
| FSIQ |  |  |  |  |  |
| Adjusted mean differences (95% CI) ^a^ | -0.23(-0.54, 0.07) | Reference | -1.51(-3.46, 0.44) | -1.30(-2.92, 0.31) | -1.69(-4.30, 0.93) |
| *P* value for linear trend | 0.137 | 0.110 | | | |
| Total behavioural problem |  |  |  |  |  |
| Adjusted mean differences (95% CI) ^a^ | 0.46(-0.08, 0.99) | Reference | 2.59(-0.56, 5.73) | 5.22(1.24, 9.20) | 7.03(0.93, 13.14) |
| *P* value for linear trend | 0.096 | 0.008 | | | |
| Internalizing behavioural problem |  |  |  |  |  |
| Adjusted mean differences (95% CI) ^a^ | 0.07(-0.11, 0.24) | Reference | 0.45(-0.62, 1.52) | 1.14(-0.25, 2.53) | 2.13(0.16, 4.10) |
| *P* value for linear trend | 0.448 | 0.044 | | | |
| Externalizing behavioural problem |  |  |  |  |  |
| Adjusted mean differences (95% CI) ^a^ | 0.08(-0.07, 0.24) | Reference | 0.59(-0.35, 1.54) | 1.40(0.26, 2.54) | 1.14(-0.64, 2.93) |
| *P* value for linear trend | 0.295 | 0.054 | | | |

Abbreviation: CI, confidence interval; SD, standard deviation.

^a^The adjustments included parental education and occupation, maternal age, maternal parity and mid-upper arm circumference, randomized regimens, birthweight for gestational age z score, household wealth at early adolescence, and adolescent sex, height-for-age z score, school type and total behavioural problem (for cognitive outcome) or FSIQ (for emotional and behavioural outcome).

Supplementary Figure 1 Flowchart.


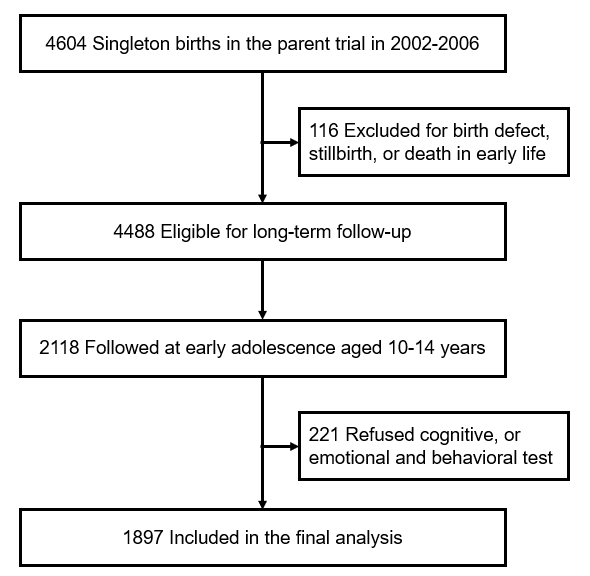


Supplementary Figure 2 Restricted cubic splines analysis for the possible non-linear relationships between maternal age at pregnancy and adolescent cognitive development and emotional and behavioural problems in a birth cohort in rural western China.


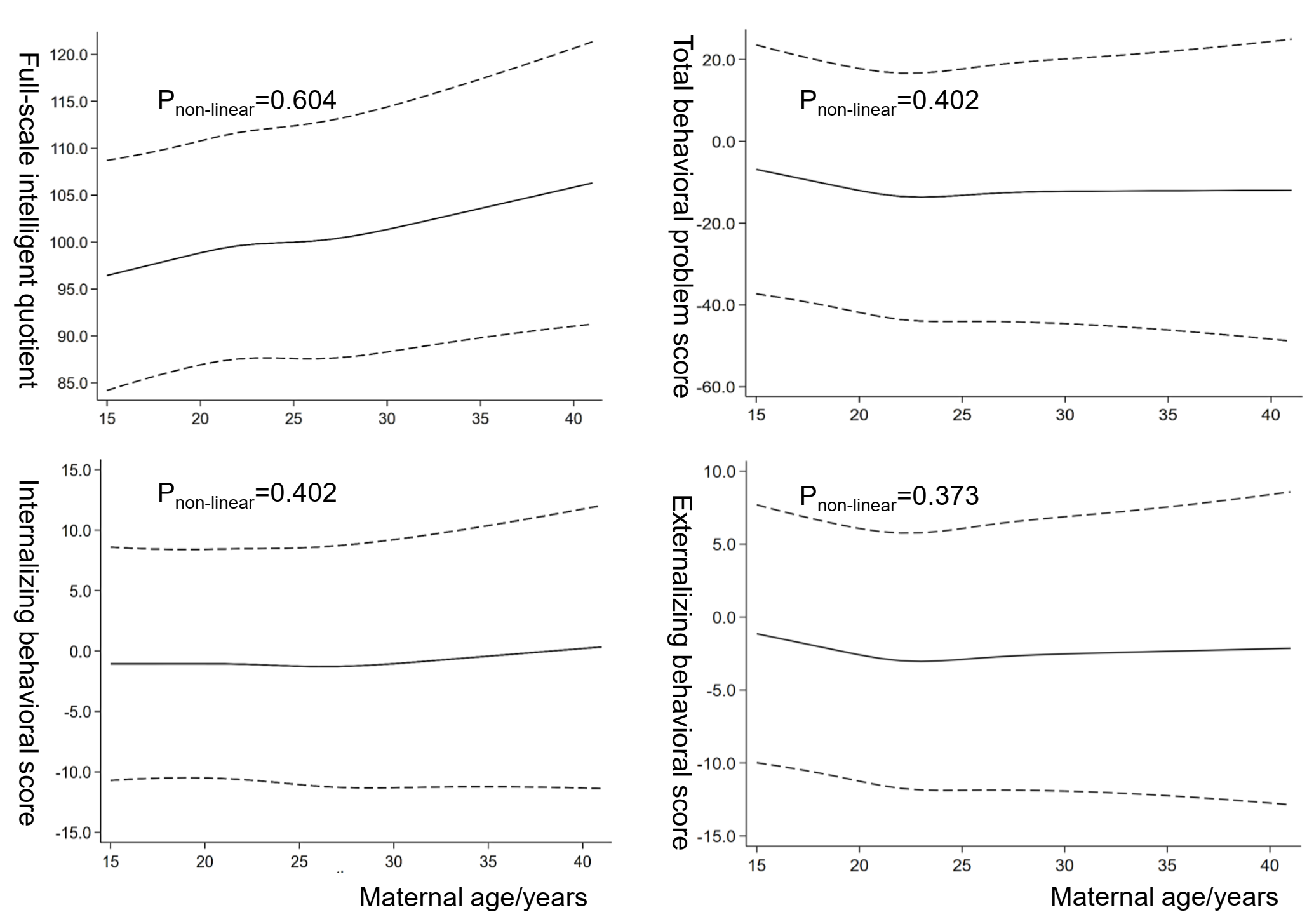


Supplementary Figure 3 Restricted cubic splines analysis for the possible non-linear relationships between paternal age at pregnancy and adolescent cognitive development and emotional and behavioural problems in a birth cohort in rural western China.


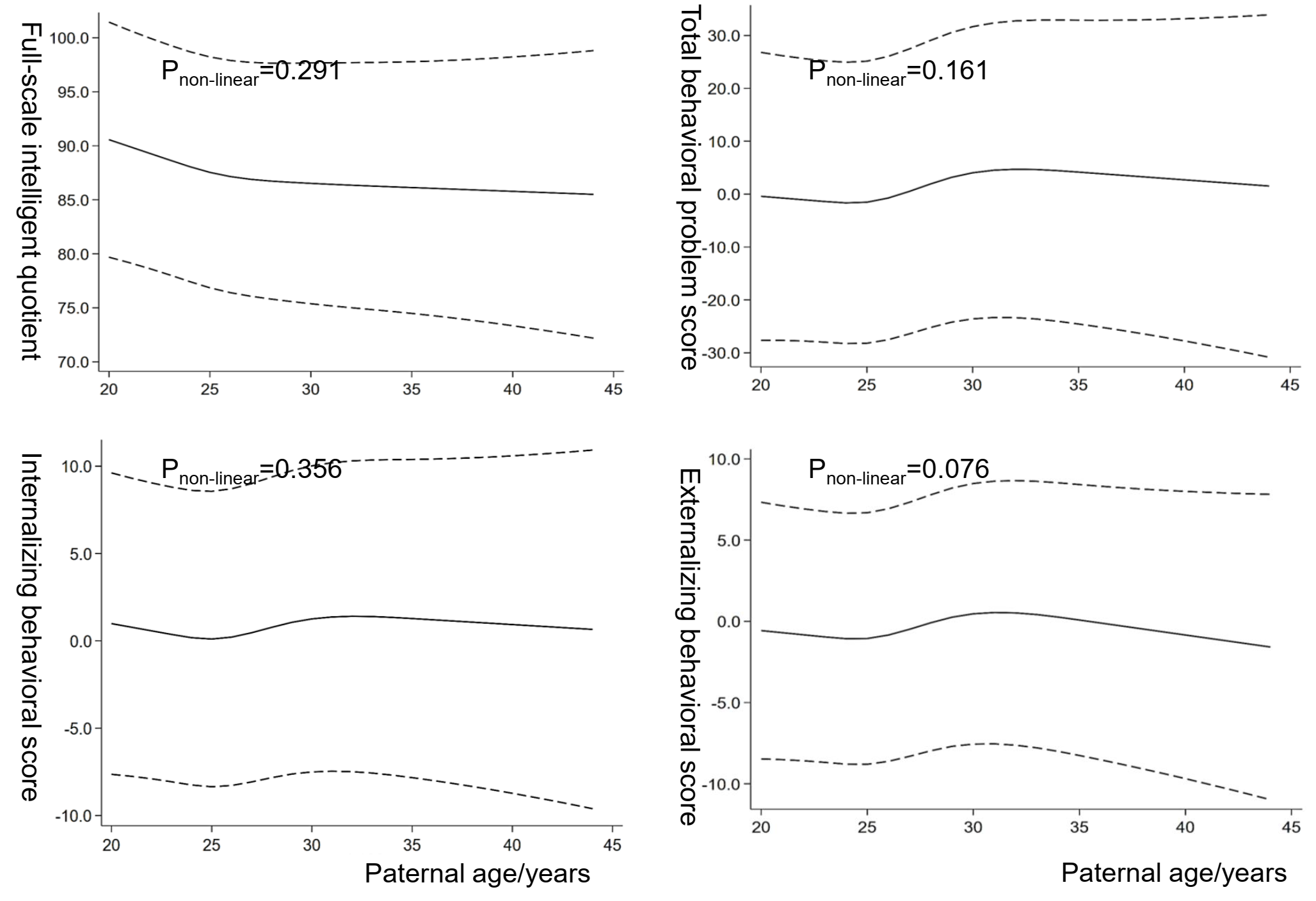

Supplement: Supplementary file 1 — Supplementary Material 1 [file 12889_2024_18309_MOESM1_ESM.docx]
